# Knowledge and attitude of human monkeypox among university students and staff in Tehran, Iran

**DOI:** 10.3389/fpubh.2025.1510185

**Published:** 2025-04-10

**Authors:** Yousef Alimohamadi, Mojtaba Sepandi, Tahereh Marhamati

**Affiliations:** ^1^Health Research Center, Life Style Institute, Baqiyatallah University of Medical Sciences, Tehran, Iran; ^2^Najmiyeh Specialized and Subspecialized Hospital, Baqiyatallah University of Medical Sciences, Tehran, Iran

**Keywords:** knowledge, attitude, human monkey pox, university students, staff, Iran

## Abstract

**Background:**

Effective control of any disease, including Monkey pox (mpox), is highly dependent on public knowledge and adherence to preventive measures. This cross-sectional study was conducted with the aim of investigating the knowledge of students and staff of Baqiyatallah University about the origin of mpox and its symptoms, transmission, prevention, and management.

**Methods:**

In the current cross-sectional study, the data was collected from Aug 5, 2024 to Sep 5, 2024. The target population consisted of students, teachers, and the staff of Baqiyatallah University, aged 18 or above. Multiple Logistic regressions were employed to assess the association between participants’ overall knowledge about human mpox as well as their attitude and their demographic data. Data was analyzed using IBM SPSS statistics version 23.0 (IBM Corp., Armonk, NY, USA) and subsequently coded and labeled. The statistical significance level was set at 5%.

**Results:**

The overall mean score of participants’ knowledge and attitude was 6.37 ± 2.20 and 4.24 ± 1.71, respectively. Based on Bloom’s cut-off points, 458 (84.3%), 78 (14.4%), and 7 (1.3%) of the participants had low, moderate, and high knowledge levels, respectively and, 279 (51.4%), 221 (40.7%), and 43 (7.9%) of the participants had low, moderate, and high attitude levels, respectively. Logistic regression analysis showed that job status(OR: 9.6, 95% CI: 1.3–66.4), major(OR: 4.3, 95% CI: 1.3–14), and higher educational level (OR: 7.6, 95% CI: 1.03–61.8), was significantly associated with participants’ knowledge about mpox.

**Conclusion:**

This study showed that university students and staff in Iran do not have enough knowledge about mpox, including its symptoms, preventive measures, and treatment, with a good understanding of local and international health authorities in the control of emerging mpox. People with higher education tend to have better knowledge, which indicates that access to reliable information sources is necessary to acquire accurate knowledge.

## Introduction

Monkeypox (mpox) is a common zoonotic infection in humans that may lead to severe illness and even death in some cases, especially in immunocompromised individuals or those with underlying medical conditions (1). This disease is endemic in parts of Central and West Africa (2). The causative agent of the disease is a virus belonging to the genus Orthopoxvirus of the same family (3). The overall Case Fatality Rare (CFR) of mpox has been estimated to be 0.62–9.51% during 1970–2022 (4). There are two types of mpox-causing viruses: clade I and clade II. Clade I is responsible for the current increase in cases in Central and East Africa (5). Historically, clade I is more virulent and causes more severe disease than clade II (6, 7). Clade II is the type that caused the global outbreak that began in 2022. Clade II is endemic to West Africa. In May 2022, outbreaks of mpox appeared suddenly and rapidly across Europe, the Americas, and then all six World Health Organization (WHO) regions. The global outbreak has mainly (but not exclusively) affected homosexuals, bisexuals, and other men who have sex with men, and has spread from person to person through sexual networks (8). In 2022, an outbreak of mpox due to clade I occurred in refugee camps in the Republic of Sudan. As of 2022, an increase in mpox cases and deaths has also been observed in the Democratic Republic of the Congo. In some regions of the country, a new branch of clade I called clade Ib is spreading from person to person (9). Symptoms of mpox include fever, chills, headache, muscle aches, and a skin rash that usually starts on the face and spreads to other parts of the body (10–12). Although the symptoms of mpox are similar to smallpox, it is generally milder. Also, lymphadenopathy, the swelling of the lymph nodes, which is a characteristic of mpox, is not seen in smallpox patients (13). In severe cases of mpox, the skin lesions can spread to different parts of the body, including the mouth, cornea, and even the genitals. However, in mild cases, the skin lesions are limited to the limbs only. The incubation period of the disease is between 5 and 21 days, and its symptoms last up to 4 weeks (14–16). Mpox usually occurs during close contact with respiratory secretions and skin lesions (17, 18). This sudden increase in morbidity and mortality caused concern to the experts of the WHO (19) so on July 23, 2022, the disease was declared by the WHO as an international public health emergency (20). Effective control of any disease, including mpox, is highly dependent on public knowledge and adherence to preventive measures (21). Therefore, it is important to improve the understanding of the epidemiology of the disease, including symptoms, transmission routes, and preventive measures (22, 23).

The first confirmed case of the disease in Iran was observed on Aug 2022 in a woman infected by her husband who had a history of traveling to Canada (24). Iran with a strategic location in the north of the Persian Gulf, is exposed to a possible mpox outbreak. This vulnerability is exacerbated by the continuous influx of migrants and refugees from Afghanistan and Pakistan (25). The confirmation of mpox cases in Pakistan has intensified concerns (26). Pakistan has a massive number of flights coming from the regions where mpox is on rise (27). On the other hand, among the Persian Gulf countries, Iran has extensive trade relations with the United Arab Emirates (28), which reported 16 mpox cases up to December 2023 (29). In other neighboring countries of Iran there have been cases of mpox (29). Despite the existence of the infectious disease monitoring system in Iran and the relatively acceptable potential of the country’s health system (30), since public participation in effective preventive measures is significantly influenced by people’s understanding of the epidemiology of the disease (31), the evaluation of the public level of society general knowledge and understanding of mpox will be essential and important.

As far as our searches show, many previous studies on mpox have focused on general populations or specific groups such as healthcare workers, but comprehensive research on university students and staff as an important population has received less consideration. In addition their findings may not align with the cultural and social context of Iran. University students and staff, as an educated and influential group in society, play a substantial role in disseminating accurate information toward infectious diseases. Therefore, assessing the knowledge and attitudes of this group is necessary. This cross-sectional study was conducted to investigate the knowledge of students and staff of Baqiyatullah University about the origin of mpox and its symptoms, transmission, prevention, and management.

## Methods and materials

The design of the current study was cross-sectional. Data were collected from Aug 5, 2024 to Sep 5, 2024. The target population consisted of students, teachers, and the staff of Baqiyatallah University, aged 18 or above. Participation in the study was voluntary. Ethical approval was obtained from the ethical committee at Baqiyatallah University (Ref. No.: IR.BMSU.BAQ.REC.1403.125).

### Sample size determination

The sample size was estimated using the following: *n* = P × (1−P) × z^2^/d^2^, where n is the sample size, *z* = 1.96 at 95% confidence level, d (0.05) is the margin of error, *p* (0.5) is an estimation of poor knowledge about the symptoms and transmission. The required sample size was estimated to be 384 participants. In order to increase the statistical power of the study a sample size of 600 was randomly selected.

### Data collection

A pre-designed questionnaire regarding the research background and study objectives was used. The questionnaire had three sections. The first section gathered demographic data. The second section evaluated participants’ knowledge of mpox. “I do not know” or incorrect responses received a score of 0, while correct answers received a score of 1. The overall knowledge score was calculated out of 15. Respondent’s attitude toward mpox was assessed through a Likert scale (Agree, Disagree, and I have no Idea) and the source of their information was also assessed in the last section. The overall knowledge score was classified into high (12–15, 80–100%), moderate (9–11, 60–79%), and low knowledge scores (0–8, ≤ 59.0%). The overall attitude score was also classified accordingly. A pilot questionnaire was performed with a small group of 30 participants to assess the reliability. The validity of the questionnaire was assessed using Lawshe’s method. The content validity ratio (CVR) for all of the items was calculated to be 0.81. The final content validity Index (CVI) of the questionnaire was 0.86.

### Statistical analysis

Categorical variables were presented as frequency and percentage. Numerical variables were presented as mean and standard deviation. Multiple Logistic regressions were employed to assess the association between participants’ overall knowledge about human mpox as well as their attitude and their demographic data. The effect size, indicated by the odds ratio (OR). To ensure the robustness of the logistic regression model, potential confounding variables were carefully considered and adjusted for in the analysis. These included age and gender, which are known to influence health literacy and access to information. The confidence intervals (CIs) reported for the ORs provide important insights into the precision of the estimates. Data was analyzed using IBM SPSS statistics version 23.0 (IBM Corp., Armonk, NY, USA) and subsequently coded and labeled. The statistical significance level was set at 5%.

## Results

**Figure 1:**
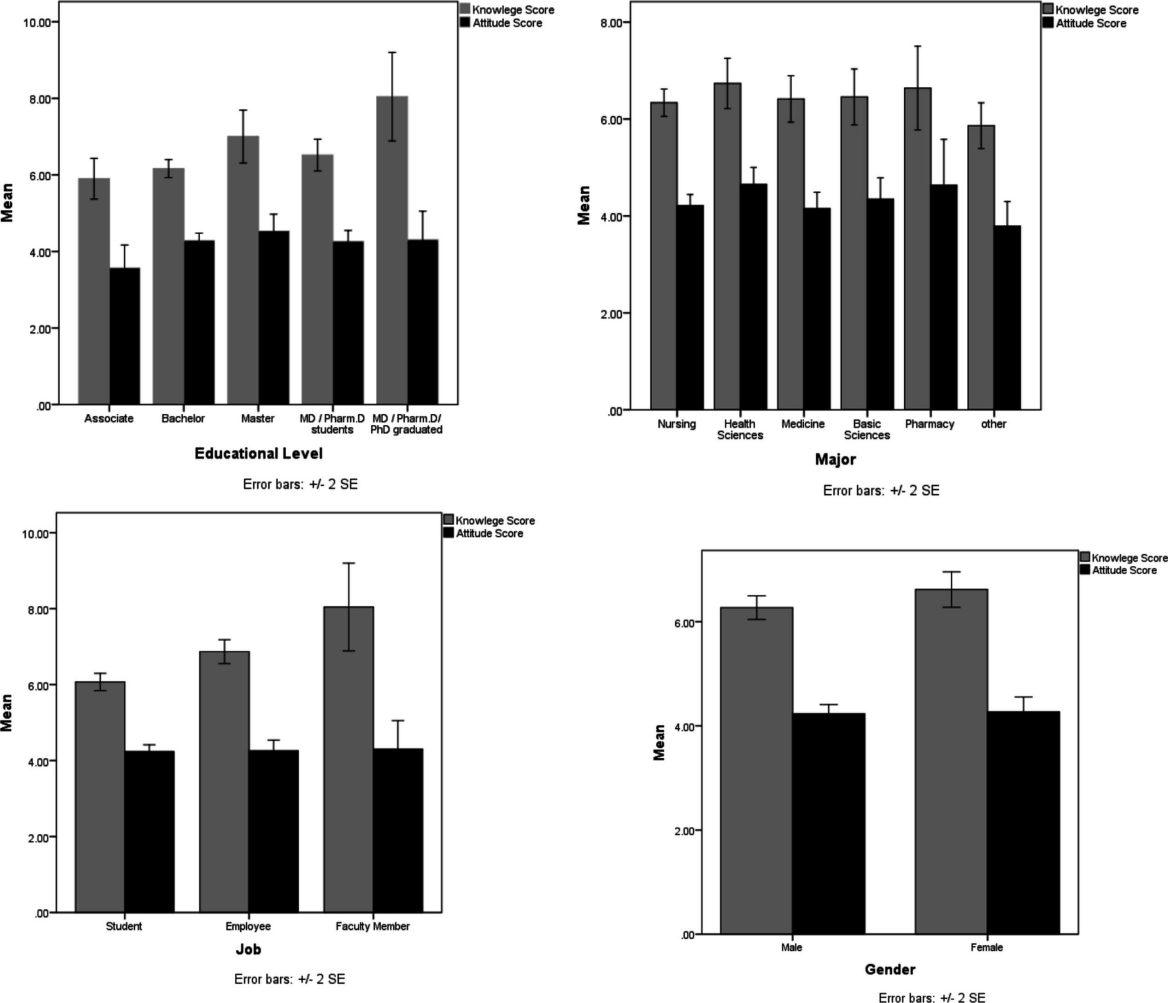
Distribution of participants’ knowledge and attitude toward human mpox across different demographics.

**Figure 2:**
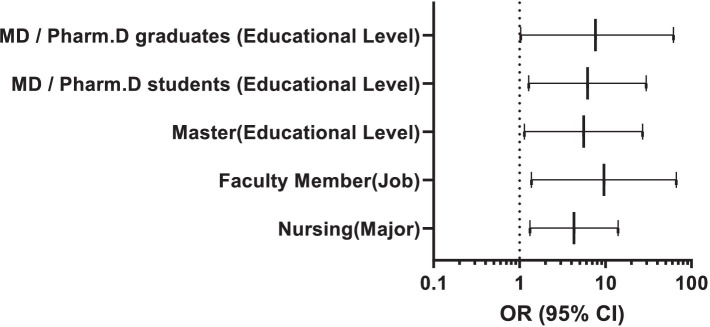
Forest plot for significant factors associated with the good and moderate knowledge about human mpox.

In this study, the response rate was 90.5%. Therefore, 543 out of 600 individuals completed the questionnaire, during the study period. As shown in Table 1, the respondents had a mean age of 27.56 ± 10.05 years, and a significant portion of them were male (68.7%, *n* = 373) and university students (67.6%, *n* = 367). Regarding mpox information sources, most of the respondents (59.5%, *n* = 323) mentioned social media as the primary information source, followed by University (18.4%, *n* = 100). Respondents’ awareness and attitude were reported in Tables 2, 3, respectively. Only 16.4% of participants gave the correct answer to the question related to the incubation period of the disease. The majority of the respondents (88.4%, *n* = 480) correctly identified that a virus causes the disease. Regarding disease transmission, over half of the respondents correctly answered the question. In terms of the symptoms of mpox, only a small proportion of participants (38.4%, *n* = 189) were aware that about one-third of cases of mpox may be without clinical symptoms, and also, only 35.7% (*n* = 194) knew that lymphadenopathy differentiates between smallpox and mpox. The majority (58.0%, *n* = 315) of participants correctly identified the mode of transmission for mpox (Table 2). Participants’ attitudes regarding human mpox were also examined and reported in Table 3. Also, 59.9% of the participants agreed that the Iranian Ministry of Health can control a probable future epidemic of mpox in the country. On the other hand, more than 53% of the respondents believed that mpox could also spread in Iran. More than half (61.3%, *n* = 333) of the participants believed that mpox can impose a lot of costs on the health care system of the country. More details are shown in Table 3. The respondents’ knowledge about mpox infection was also analyzed quantitatively. The overall mean score of participants’ knowledge and attitude was 6.37 ± 2.20 and 4.24 ± 1.71, respectively. The comparison of mean scores of knowledge as well as the attitude among subgroups is shown in Table 4. There was a statistically significant difference between the occupational groups in terms of the average knowledge score. So the highest score was related to faculty members (8.04 ± 2.77) and the lowest score was related to students (6.06 ± 2.18) (*p* < 0.0001). There was a significant relationship between the educational level of participants and knowledge. So the highest score was seen in participants with higher scientific degrees (8.04 ± 2.77) and the lowest score was related to Associates (5.89 ± 1.66) (*p* < 0.0001). Regarding the attitude score, there was a significant relationship between the major of participants and attitude. The highest score of attitude was observed in the fields of health sciences and pharmacy, respectively (*p* = 0.04). The study also examined the relationship between demographic factors and participants’ knowledge as well as an attitude toward mpox. (Table 4; Figure 1). Table 5 shows the analysis of factors associated with the (Good and Moderate) knowledge and attitude about mpox. Based on Bloom’s cut-off points, 458 (84.3%), 78 (14.4%), and 7 (1.3%) of the participants had low, moderate, and high knowledge levels, respectively and, 279 (51.4%), 221 (40.7%), and 43 (7.9%) of the participants had low, moderate, and high attitude levels, respectively (Data not be shown in the table). Logistic regression analysis showed that job status, major, and educational levels were significantly associated with participants’ knowledge about mpox. However, the logistic regression analysis revealed that only major was significantly associated with respondents’ attitude toward mpox (Table 5; Figure 2).

**Table 1:** Number and percentages of the questions on demographics (*n* = 543).

Demographic	Groups	Frequency	Percentage
Age	27.56 ± 10.05		
Gender	Male	373	68.7
	Female	170	31.3
Job	Student	367	67.6
	Employee	153	28.2
	Faculty Member	23	4.2
Major	Nursing	232	42.7
	Health Sciences	83	15.3
	Medicine	101	18.6
	Pharmacy	11	2
	Basic Sciences	55	10.1
	Other	61	11.2
Educational level	Associate	39	7.2
	Bachelor	308	56.7
	Master	49	9
	MD/Pharm. D student	124	22.8
	MD/Pharm. D/PhD graduates	23	4.2
Is it necessary to hold short-term educational programs about pox at the university level?	Yes	402	74
	No	74	13.6
Source of information about human mpox	TV	38	7
	Social media	323	59.5
	University	100	18.4
	Other	44	8.1

**Table 2:** Knowledge assessment (Item-wise) of mpox (*n* = 543).

Knowledge assessment	Choices	The correct answer, *n* (%)
1. Cause of mpox in humans?	Fungal, parasite, bacteria, virus, None of the above	480 (88.4)
2. Discovering the virus for the first time.	Monkeys, Central Asia, US, None of the above	190 (35.0)
3. Infection is transmitted through	From animal to human, From a person to another person, breathing path, All of the above	361 (66.5)
4. Infection is transmitted	From the pregnant mother to the fetus, clothing, long face-to-face exposure, and All the above	315 (58.0)
5. Signs and symptoms of mpox	All infected cases have clinical symptoms, all infected cases are without clinical symptoms, about one-third of cases may be without clinical symptoms, and about 80% of cases are without clinical symptoms	189 (38.4)
6. The incubation period for the virus	5–13 days, 3–7 days, 5–21 days, I do not know	89 (16.4)
7. Clinical Manifestation of mpox compared to smallpox	More severe, milder, similar, I do not know	197 (36.3)
8. In mpox, how long does the rash usually appear after the onset of fever and lymphadenopathy?	2 to 5 days, 1 to 3 days, a week, immediately	189 (34.8)
9. Patients who have been vaccinated against smallpox	They have fewer lesions than unvaccinated people, smallpox vaccination has no effect on the lesions caused by mpox, small pox vaccination may intensify the lesions caused by mpox, None of the above.	256 (47.1)
10. Lymphadenopathy	It is a significant clinical feature that distinguishes it from smallpox, It is also present in smallpox, but It is present only in smallpox	194 (35.7)
11. How long is the mpox patient infectious?	As long as the antibody is detectable in the blood, Until the new skin forms, Until the person feels healed, None of the above.	210 (38.7)
12. Can mpox be a potential threat (like COVID-19) on a global scale in the future?	Probably, never, in the absence of universal vaccination; yes, I do not know	165 (30.4)
13. The fatality rate (prognosis) of mpox	It is more than 50%., It is less than 10%, It is not fatal at all, I do not know	210 (38.7)
14. Reservoir of mpox	The definitive animal reservoir has not been identified, Rodents, Monkeys, The first and third options are correct	196 (36.1)
15. What should be done if a person comes into unprotected contact with a case of mpox?	No special action is required, Monitor for 3 weeks after the first symptom, regardless of symptoms must be monitored for 3 weeks after the last contact, Monitor for symptoms such as fever, chills, rash, and lymphadenopathy for 3 weeks after the last contact	223 (41.1)

**Table 3:** Attitude assessment (item-wise) of mpox (*n* = 543).

Attitude assessment	I agree	I have no opinion	I disagree
1. I am confident that mpox will be controlled worldwide.	300 (55.2)	152 (28.0)	90 (16.6)
2. I am sure that the Ministry of Health and relevant experts can control it in case of possible epidemic in Iran	325 (59.9)	152 (28.0)	65 (12.0)
3. I have a bad feeling about mpox and I think it could turn into a pandemic.	175 (32.2)	192 (35.4)	172 (31.7)
4. Mpox can impose a lot of costs on the health care system of the country.	333 (61.3)	145 (26.7)	64 (11.8)
5. It is not possible for mpox to spread in Iran.	106 (19.5)	147 (27.1)	289 (53.2)
6. Mass media cannot play an influential role in preventing mpox in society.	127 (23.4)	85 (15.5)	329 (60.6)
7. I need to get more information about mpox and other emerging diseases.	408 (75.1)	96 (17.7)	38 (7.0)
8. Travel to mpox endemic areas should be forbidden.	190 (35.0)	200 (36.8)	152 (28.0)

**Table 4:** Participants’ knowledge and attitude toward human mpox according to demographics.

	Knowledge			Attitude		
Demographic	Mean ± SD	Median (Q3-Q1)	*p*-value	Mean ± SD	Median (Q3-Q1)	*p*-value
Gender						
Male	6.26 ± 2.18	6 (8–5)	0.08	4.23 ± 1.66	4 (5–3)	0.82
Female	6.61 ± 2.21	6 (8–5)		4.27 ± 1.84	4 (6–3)	
Job						
Student	6.06 ± 2.18	6 (7–5)	0.0001	4.23 ± 1.72	4 (5–3)	0.97
Employee	7.02 ± 2.10	7 (8–6)		4.26 ± 1.71	4 (5–3)	
Faculty Member	8.04 ± 2.77	8 (11–5)		4.30 ± 1.79	4 (6–3)	
Major						
Nursing	6.33 ± 2.15	6 (8–5)	0.27	4.21 ± 1.72	4 (6–3)	0.04
Health Sciences	6.73 ± 2.36	6 (8–5)		4.65 ± 1.58	5 (6–4)	
Medicine	6.44 ± 2.47	6 (8–5)		4.21 ± 1.67	5 (5–3)	
Pharmacy	6.63 ± 1.42	6 (7–6)		4.63 ± 1.56	5 (6–3)	
Basic Sciences	6.45 ± 2.14	7 (8–5)		4.34 ± 1.63	4 (5–3)	
Other	5.83 ± 1.76	6 (7–4.75)		3.70 ± 1.93	4 (5–2)	
Educational level						
Associate	5.89 ± 1.66	6 (7–5)	0.0001	3.56 ± 1.88	3 (5–2)	0.10
Bachelor	6.16 ± 2.06	6 (8–5)		4.27 ± 1.74	4 (6–3)	
Master	6.97 ± 2.42	7 (9–5)		4.53 ± 1.54	5 (6–4)	
MD/Pharm. D students	6.51 ± 2.31	6 (8–5)		4.25 ± 1.61	5 (5–3)	
MD/Pharm. D/PhD graduated	8.04 ± 2.77	8 (11–5)		4.30 ± 1.79	4 (6–3)	

**Table 5:** Multiple analysis of factors associated with the good and moderate knowledge and attitude about human mpox.

Factors	Knowledge				Attitude			
	OR	LCI	UCI	*p*-value	OR	LCI	UCI	*p*-value
Gender								
Male	1				1			
Female	0.84	0.46	1.55	0.59	1.15	0.75	1.76	0.50
Job								
Student	1				1			
Employee	0.95	0.38	2.34	0.91	1.33	0.68	2.57	0.39
Faculty member	**9.60**	**1.38**	**66.47**	**0.02**	1.16	0.32	4.25	0.81
Major								
Nursing	**4.31**	**1.32**	**14.00**	**0.01**	1.28	0.68	2.40	0.43
Health Sciences	2.75	0.80	9.39	0.10	**2.23**	**1.08**	**4.61**	**0.03**
Medicine	1.86	0.43	8.04	0.40	2.13	0.71	6.37	0.17
Pharmacy	1.15	0.94	14.12	0.91	3.71	0.73	18.82	0.11
Basic Sciences	2.48	0.65	9.47	0.10	1.08	0.50	2.34	0.83
Other	1				1			
Educational level								
Association	1				1			
Bachelor	3.02	0.68	13.43	0.14	1.43	0.71	2.87	0.31
Master	**5.56**	**1.14**	**26.98**	**0.03**	1.80	0.75	4.28	0.18
MD/Pharm. D students	**6.18**	**1.28**	**29.71**	**0.02**	1.75	0.75	3.90	0.16
MD/Pharm. D/ PhD graduates	**7.66**	**1.03**	**61.89**	**0.04**	1.01	0.24	4.07	0.99

*p-values less than 0.05 were considered statistically significant.*

OR, Odds ratio; LCI, Lower limit confidence interval; UCI, Upper limit confidence interval. The statistically significant values are shown in bold.

## Discussion

The findings of this study reveal significant gaps in knowledge about mpox among university students and staff in Iran, which reflect broader global public health challenges in addressing emerging infectious diseases. The phenomenon of globalization, characterized by increased international travel, trade, and migration, has amplified the risk of disease transmission across borders (32). Iran’s geographical location, extensive trade relations, and the influx of migrants and refugees from neighboring countries such as Afghanistan make it particularly vulnerable to outbreaks of diseases like mpox. These factors are not unique to Iran. In fact across the globalized world, infectious diseases can rapidly spread from endemic to non-endemic regions, as seen with mpox outbreaks in Europe, North America, and other parts of the world in 2022–2023 (13). For instance, the initial spread of mpox outside Africa was largely attributed to international travel and close-contact networks, highlighting the role of globalization in disease transmission (32). Iran, a country of more than 80 million people in the north of the Persian Gulf and the Strait of Hormuz, connected to the Arab, European, and Asian markets (33), is exposed to a possible mpox outbreak. The first confirmed case of the disease in Iran was observed on Aug 2022 in a woman infected by her husband who had a history of traveling to Canada (24). This vulnerability is exacerbated by the continuous influx of migrants and refugees from Afghanistan and Pakistan (25), as countries that lack effective monitoring systems (34, 35). Afghanistan is poorly placed to address infectious outbreaks, including mpox outbreaks (36). The length of the common border between Iran and Afghanistan is about 900 kilometers (37). It is very difficult to control such a long border. Unofficial statistics indicate that in 2024, more than 14 million Afghan refugees were living in Iran, of which only 2 million were registered as legal immigrants, and the rest were living in Iran illegally. Unfortunately, these immigrants are smuggled to and from Afghanistan (38). This high volume of immigrants and the long borders between the two countries, along with the lack of an efficient health care system in the country of origin of immigration, increases the potential for disease outbreaks in Iran. The confirmation of mpox cases in Pakistan has intensified concerns (26). The rapid deportation of Afghan refugees from Pakistan heightens fears of further virus transmission (26). The length of the common border between Iran and Pakistan is also about 900 kilometers (39). Pakistan has a massive number of flights coming from the regions where mpox is on rise (27). The lack of a specific vaccine in Iran and Afghanistan further complicates the situation, underscoring the need for global collaboration in vaccine distribution and equitable access to medical resources (36). On the other hand, among the Persian Gulf countries, Iran has extensive trade relations with the United Arab Emirates (28), which reported 16 mpox cases up to December 2023 (29). In other neighboring countries of Iran [Saudi Arabia (8 cases), Qatar (5 cases), Oman(3 cases)and Bahrain(2 cases)], there have been cases of mpox (29).

This study showed that the participants had poor knowledge about the mpox while a positive attitude toward mpox prevention. Participants were also concerned about the outbreak of mpox. This finding is concerning as it highpoints potential gaps in the knowledge needed for management of mpox cases. Insufficient knowledge can lead to interruptions in detection and response, contributing to the spread of the disease.

Our results also showed that job status, major, and education levels are significant predictors of knowledge. Indeed, faculty members who were exposed to scientific journals had greater odds of having a higher level of knowledge compared to that were less exposed to scientific journals. This finding is not unexpected in view of the fact that one can benefit from updated access to accurate and correct information. Also postgraduate education and major as nursery were associated with higher knowledge. Given that no specific training has been conducted for any major, nursing majors have been observed to possess a better knowledge of mpox in multiple analyses. This result may deserve further investigation. Meanwhile, the participants in the present study were students and employees of one of the universities of medical sciences. This is worrisome because it is likely that the general public’s level of awareness is lower than this. Public knowledge about mpox was evaluated in different countries and similar findings were reported (40–42). Alshahrani et al. (40) assessed knowledge of monkeypox viral infection among the general population in Saudi Arabia, findings revealed Participants with higher education levels and healthcare backgrounds demonstrated significantly better knowledge about mpox. Gallè et al. (41) evaluated the awareness and knowledge of monkeypox (mpox) among Italian adults during the early stages of the 2022 outbreak. Results indicated low to moderate levels of knowledge about mpox, with many participants unaware of key aspects such as transmission routes, symptoms, and prevention (41). Crosato et al. (42) studied the perception, awareness, and vaccination acceptance of monkeypox (mpox) among an at-risk population in Brescia, Italy. Findings revealed moderate levels of awareness about mpox, with many participants aware of the disease but lacking detailed knowledge about its transmission and prevention (42).

Launching awareness campaigns in the general population seems necessary. A recent study of healthcare workers in several countries in the Persian Gulf region and the Middle East showed that most healthcare professionals have moderate knowledge about the mpox virus (43). One possible explanation is that with the reduction of importance of some infectious diseases during past decades, such as smallpox, teaching these diseases is neglected in the learning resources of medical sciences. This emphasizes the need to revise the educational resources along with the implementation of health education programs as well as providing continuing medical education (CME) on emerging and reemerging diseases for health professionals and university staff. In addition, launch awareness campaigns in the general population is inevitable for the prevention or control possible outbreaks in the future (42). As formerly shown during COVID-19 pandemic, training and awareness assessment is essential (44).

Most participants in the present study were aware of the possibility of human-to-human transmission of the disease. However, most of the participants were not fully aware of the modes of transmission of the disease, its symptoms and complications. A similar finding has been obtained from studies conducted in developing countries (22, 45). Awoyomi et al. (22) explored the perceptions and knowledge of mpox (monkeypox) among critical stakeholders in Nigeria, including healthcare workers, policymakers, and community leaders. Findings revealed significant gaps in knowledge about mpox, particularly regarding its transmission routes, symptoms, and preventive measures. Many stakeholders lacked awareness of the zoonotic origins of the disease and its potential for human-to-human transmission. Halboup et al. (45) in Yemen reported low levels of knowledge about mpox among the general population, with many participants unaware of the disease’s symptoms, transmission routes, and preventive measures.

The present study, consistent with previous studies (40, 46), showed that participants were somewhat familiar with the skin symptoms of mpox. This may be due to the visual learning approach through watching TV and other social media. According to the relationship between general knowledge about mpox and the socio-demographic data of the participants, people with higher education had higher knowledge scores. This result was predictable, as these individuals are expected to have reliable access to information, participate in reading scientific publications, and have the skills to evaluate information sources and make informed decisions about their quality. Similar findings were obtained in a recent survey conducted in middle eastern countries, where education level and age were the influencing factors on mpox awareness (22).

The study’s findings also align with global concerns about the role of misinformation and infodemics in public health. The reliance on social media as a primary source of information about mpox is a trend observed worldwide, as seen in studies from Jordan, Iraq, Saudi Arabia, (40, 47, 48), the Philippines (49), and Yemen (45) While social media can be a powerful tool for disseminating information, it also poses significant risks due to the spread of misinformation. Abu-Farha et al. (48) in Jordan reported that social media was identified as the primary source of information, which also led to the spread of misinformation and misconceptions. Berdida (49) assessed the knowledge of human monkeypox (mpox) among the general population in the Philippines reported that social media was the primary source of information about mpox, but it also contributed to the spread of misinformation and misconceptions. Halboup et al. (45) in Yemen reported the role of social media as a primary source of information, which also contributed to the spread of misinformation.

The COVID-19 pandemic demonstrated how infodemics can undermine public health efforts, leading to harmful behaviors and eroding trust in health authorities. Addressing this issue requires a coordinated global response, including promoting digital health literacy, combating misinformation, and leveraging social media to disseminate accurate information from reliable sources.

The study’s results also highlight the critical role of education in shaping public knowledge and attitudes toward emerging infectious diseases. Higher levels of education were associated with better knowledge about mpox, likely due to greater access to reliable information and critical thinking skills. However, the study’s participants—university students and staff—likely represent a more educated segment of the population, suggesting that knowledge gaps may be even more pronounced among the general public. This underscores the need for targeted educational campaigns that reach diverse populations, including those with lower levels of education and limited access to reliable information.

Based on the results of the present study, social media is considered a primary source of information about mpox. Those with higher education are better at seeking online information. Consequently, differences in media use habits and electronic health literacy skills affect the amount and quality of information. Public health organizations should know that education is a means of acquiring knowledge and a vital factor in shaping how people interact with the world and understand information. Therefore, they should not be satisfied with only the dissemination of information. Rather, it is essential to focus on ability to use this information effectively. Another extremely important issue is the infodemic and misinformation which could have a tremendous impact on the quality of people’s awareness. The trend of relying on social media for health information accelerated during the COVID-19 pandemic, most likely due to quarantines and social distancing rules at the time, which led many people to rely on social media and the internet for information (50). So, infodemic crises must be considered as a priory of health decision making authorities in the country. We warn our society about misinformation distributed among public in the form of news, recommendation and guidelines about mpox. During the COVID-19 pandemic in Iran and other countries in the region, the situation of managing information resources in cyberspace was not favorable, so that misinformation caused several deaths by direct consumption of alcohol and detergents to avoid the risk of coronavirus (51). The spread of misinformation about diseases may has political, economic and health related aspects which require precise strategies for dealing. The health authorities of the country should accept this issue and plan for it because cyberspace is usually not a reliable source of health-related information. Therefore, it is necessary to promote public awareness about the potential risks of relying on social media for health-related information and encourage the use of reliable sources of information such as health care providers and the official website of the Ministry of Health and universities.

The present study showed that the university is the second most common source of information about mpox. This finding can be explained by the fact that the respondents were academics. Consistent with similar studies (45, 47, 48) most respondents in the current study showed high levels of confidence in the ability of Ministry of health professionals to control mpox. Halboup et al. (45) assessed the knowledge and perceptions of the Yemeni public regarding the emerging human monkeypox (mpox) outbreak and highlighted the role of social media as a primary source of information, which also contributed to the spread of misinformation. Abu-Farha et al. (48) assessed knowledge and perceptions about the mpox in Jordan and concluded that social media was identified as the primary source of information, which also led to the spread of misinformation and misconceptions.

This assurance can be gained from first-hand experience during the COVID-19 pandemic, where the WHO and health authorities were able to eventually contain the epidemic (52, 53). Mpox is a serious public health threat worldwide (54). Given the significant risks and the lack of access to a vaccine, prevention strategies play an important role in minimizing the rate of infection and stopping the spread of the disease. Increasing awareness as well as adopting preventive strategies can prevent infectious diseases such as COVID-19 and influenza. Carrying out interventions and public health measures without people’s encouragement and cooperation will not have the expected efficiency.

The findings of this study have significant implications for public health interventions aimed at improving awareness and preparedness for emerging infectious diseases like mpox. Universities, as hubs of knowledge dissemination and innovation, are uniquely positioned to play a pivotal role in these efforts. Given that faculty members and postgraduate students demonstrated higher levels of knowledge, university-based interventions should leverage these groups as ambassadors to disseminate accurate information about mpox and other emerging diseases. The study’s findings also highlight the need for tailored interventions to address knowledge gaps among specific subgroups. For instance, undergraduate students and staff with lower levels of education may require more accessible and engaging educational materials, such as infographics, videos, and interactive sessions, to enhance their understanding of mpox. Additionally, collaborations between universities and public health authorities could facilitate the development of standardized training modules on emerging infectious diseases, which could be disseminated across academic institutions nationwide.

### Study strengths and limitations

The results of the present research can be of great contribution in formulating and implementing policies and intervention strategies aimed at preventing and managing emerging diseases in the country. The results of this study emphasize the existence of significant deficiencies in the amount of knowledge and reliable sources that the people of the country refer to for information about mpox. Therefore, the need for campaigns that are launched and supported by the health authorities and target the general population of Iran in order to improve their understanding of emerging diseases and promote the use of reliable information sources is strongly felt. However, the current study had several limitations; therefore, the results should be interpreted with caution. First, our study was cross-sectional in design, causality cannot be assessed. Second, there is a potential for selection bias, as Iran is neither an endemic area nor has reported a high incidence rate. On the other hand, the students, professors, and staff of a medical sciences university in the capital of Iran may not be representative of the entire Iranian population, so the findings may not be generalizable to the broader population in Iran. Therefore, our study may underestimate the level of knowledge in the general population, limiting the generalizability of the findings to the broader population. Third, this study adopted convenience sampling. The key disadvantage of convenience sampling is that the sample lacks generalizability. To address these limitations, future studies should employ more rigorous sampling methods, such as stratified or cluster sampling, to ensure a representative sample of the population. For example, studies could include participants from multiple universities across different regions of Iran, as well as individuals from non-academic settings, such as rural communities and urban workplaces. This would provide a more comprehensive understanding of mpox knowledge and attitudes across diverse demographic and socioeconomic groups.

Another important direction for future research is the exploration of cultural and contextual factors that influence knowledge and attitudes toward mpox. Qualitative studies, such as focus group discussions and in-depth interviews, could provide deeper insights into the barriers and facilitators of health literacy in different populations. For example, research could examine how cultural beliefs, stigma, or language barriers affect the uptake of accurate information about mpox and other emerging infectious diseases.

## Conclusion

This study showed that university students and staff in Iran do not have enough knowledge about mpox disease, including its symptoms, preventive measures, and treatment, with a good understanding of local and international health authorities in the control of emerging mpox. People with higher education tend to have better knowledge, which indicates that access to reliable information sources is necessary to acquire accurate knowledge. Social media is the main source of information about mpox among the public. Therefore, there is an opportunity to use this platform to disseminate accurate information. It is very important to implement educational campaigns aimed at filling knowledge gaps and correcting misconceptions to increase preparedness against possible disease outbreaks. Increasing knowledge about mpox is key to increasing the capacity to respond to mpox cases and transfer data to the disease surveillance system.

## Data Availability

The raw data supporting the conclusions of this article will be made available by the authors, without undue reservation.
